# A War Hospital Supply Depot

**Published:** 1915-12-18

**Authors:** 


### A WAR HOSPITAL SUPPLY DEPOT.

### Some Guide to its Organisation.

BY AN ADMINISTRATOR.

THIS war has opened out innumerable opportunities for women of leisure to find useful employment of a public character. It was the impulse which, happily, corresponded with the opportunity that prompted a body of women in Plymouth to organise themselves into a useful society and do something to assist the nation, the result being that the Plymouth War Hospital Supply Depôt was opened at 4 Elliot Street on June 24, 1915.

The wide field of operations, coupled with the magnitude of the war, showed great need for auxiliary help to supply surgical requirements near to the scene of fighting. Personal observations have proved that, however efficient the Government Departments were in providing hospital requirements, there were frequent emergencies where the wounded and sick had their sufferings increased by lack of the necessary materials at distances from the base hospitals. That knowledge focussed this movement, and the success has exceeded all expectations. The Plymouth depôt now has a register of over 800 women. It has been suggested that a few lines on the subject in this Journal might be of some service to other places contemplating opening depôts, and, if so, an outline of the operations at this place may perhaps form some guide to others.

The initial movement was made by an announcement in the local Press under the name of a popular and influential lady in the district. Numerous replies were attracted, which laid the foundation of the depôt. A small committee was formed, with a president, vice-president, chairwoman, superintendent and director of the work in the depôt, an hon. treasurer, and hon. secretary. Immediate steps were taken to find suitable premises. A sympathetic owner placed a suitable building at the free disposal of the committee, whereupon the whole premises were thoroughly well-cleaned, drains tested, lighting and heating attended to, all of which was voluntarily done by tradespeople who wished to assist the movement and gave their labour and materials. The equipment of the building was proceeded with on the same voluntary lines, and in no case did the promoters meet with a refusal. In fact, the local tradespeople seemed to compete with each other in their desire to help the work. The articles lent were tables (with white oilcloth to cover them), chairs, cupboards, sewing-machines, roller-bandage machines, cutting-out scissors, office furniture, and the necessary crockery for supplying tea to the workers on the premises. Such a generous response placed the depôt in the fortunate position that when it opened for work the committee had practically obtained all they required. There were also further gifts, —viz., clocks, pin-cushions, tape measures, parcel-weighing machines, and cloakroom equipment, which were all immediately accumulated.

The first step towards organisation was the election of the superintendent and director of the depôt, who, as the chief, controls the various departments and all work carried on in the building, and to whom everything in relation to all working matters has to be referred. Her decision is final on all points of daily occurrence, though, whenever necessary, she refers matters to the committee, which meet as occasion arises. She is responsible for the selection and ordering of materials, interviewing all callers and visitors, answering all queries, and explaining the method of work, and it is for her to help to keep the general machinery running smoothly. In the selection of a superintendent it is absolutely essential, as was done in this case, to obtain the services of a fully-trained practical worker. The superintendent guides and directs the following departments, keeping herself in close touch with each one of them :

1. Many-tailed bandages.

2. Roller bandages.

3. Swabs and pneumonia jackets.

4. Shirts.

5. Pyjamas.

6. Sandbags and splints.

7. Old linen.

### THE DUTY OF THE DEPUTIES.

A lady was appointed to take charge of each separate room, and to be entirely responsible for the work done in her room, having first thoroughly learnt each step of the work herself (which is a most important point). Each of these ladies then appoints and trains a certain number of her workers to act as her deputies, the rule being that one of them must always be on duty to help her or take charge of the room in her absence. Each room, then, is never left without a competent head who can instruct newcomers and keep all the workers fully employed, at the same time supervising the work, taking care that it is done up to the standard required, and when not satisfactory, insisting upon it being re-done. Further, it will be observed that much tact and patience are necessary to fill these positions successfully. Another duty of a deputy is to see that a certain amount of work is left prepared ready for her relief to begin with, and in that way to prevent any hitch in the continuous running of the work in any of the rooms. With such careful preparations as these in which the smallest details had been given close attention, the opening of the premises and the starting of operations were noticeable for the smoothness with which the work began. Large numbers of workers immediately joined the movement, and attended daily to give their labours. As already stated, over 800 are now identified with this work, and the average attendance each day is over 100, thereby keeping a steady output of work without overcrowding the premises.

It is generally understood that each worker contributes 1s. per week in order to help to purchase the materials. That payment is not compulsory, but needless to say there are very few who refrain from paying this sum, while others pay considerably more. Collection of these amounts is arranged by each worker placing her money in a box in the hall, and at the same time signing her name in a book provided, marking the payment therein. Each payment is subsequently entered by the honorary secretary in an indexed book, thereby easily showing the exact amount which has been contributed by each worker since she joined the depôt. That precaution is advisable in order to strengthen the confidence the workers may have in their officers, and also shows individual contributions to the funds. All these things soon demonstrated there was every justification in opening the depôt. The increasing daily attendances prove that the women are keen to do something for the sailors and soldiers fighting for our country. It is common knowledge that many people are anxious to work, but do not exactly know what to do or how to begin, and in such cases they are most thankful to be shown a useful method of " doing the right thing at the right time," and also to know that the product of their labour goes direct to the place where it is needed at the moment, and is not wastefully accumulated or delayed in large quantities (as is known to have been the case in transit from some sources). The rooms are opened daily (except Sundays) from ten to six, and on Saturdays from ten to one, the individual workers being free to come at any time, except the ladies in charge, or their deputies, one of whom is expected to be continuously on duty.

### The Essential Rules.

The invitation to workers and other sympathisers is "Come as often, and stay as long as you can," and many people who can only give an hour at a time find this very convenient. All workers on arrival enter their names daily in a book provided in the hall, which thereby compiles a record of the regular attenders. The rules in the institution are few. Each worker must wear an apron, a pair of sleeves, a cap, and bring her own thimble and scissors. No bags or other private articles must be placed on the work-tables. The workers "must not make knots," and they "must daily collect all pins from the floors" when the work is finished. The lady in charge of each room keeps a daily record of the work done, giving a weekly report to the superintendent, who makes a tabulated account of all work finished in the depôt. The articles are packed in the respective rooms where they are made and passed on thus completed to the packing room, where the variety of articles to make a consignment are finally grouped and dispatched to their destination. All articles appealed for from hospitals at home or abroad, ashore or afloat, are sent off direct to them at the earliest moment. There is never a refusal from this depôt, and the workers have the great satisfaction of having their consignments acknowledged by the receiver, thereby realising that the fruits of their labour are not hung up at stores or bases.

The workers come from all classes, and combine together with the heartiest goodwill and fellowship, which makes it a delightful experience to be associated with them. Many girls, hard at work in their various occupations during the week, come and give their scanty leisure, and again many workers who come very long distances bring their lunch and stay working all day, thus showing the character of the spirit animating them. Now that the winter evenings are closing in, several classes have been formed for girls to sew and knit at various places, and their work will feed the depôt with woollies, shirts, pyjamas, and articles of that character. But the articles for surgical work must only be done at the depôt, and none of that character is put out.

### The Organisation of Branch Depôts.

Soon after the depôt opened its example was effective throughout the West of England. Applications came in from towns and villages in South Devon and the County of Cornwall, asking to be accepted as branches to help with patterns and to guide and direct their preliminary operations. The staff from the Plymouth depôt gladly undertook this guidance, making only two conditions: first, that all work done should be exactly to the Plymouth patterns; and, secondly, that the Plymouth Central Depôt should not have the financial liabilities of the branches. There are now fourteen branches in the country districts, and a similar institution to the Plymouth depôt has also been formed subsequently in the adjoining town. The branches have sent in large quantities to 4 Elliot Street, numbering over 13,000 of most excellently well-finished articles, and that part of the depôt has developed so rapidly that a special department has now been formed to deal with the work from the numerous branches. In order to keep up the standard of the work done and sent from Plymouth, each article must be examined and approved before it is packed and sent out, no matter where it comes from. There are also many gifts of materials and articles from individuals which are gratefully received. This depôt has now been at work nearly five months, and at a recent date the output was 68,748 articles, which have been sent to casualty clearing hospitals and base hospitals in France and the Mediterranean, Malta, hospital ships, hospital trains, the Serbian Relief Depot, the Belgian Field Hospital, the French Red Cross, and to the British East African Force. Sandbags were also sent to the fighting lines in Flanders, France, and the Dardanelles.

On the occasion of the visit of King George and Queen Mary to Plymouth in September last, their Majesties made a tour of inspection of the various departments in the depôt. They showed their appreciation of the useful work being done there, and, needless to say, the workers were much encouraged by this compliment extended to them.

### The Financial Arrangements.

The financial side of the management is, of course, a vital one, and in this the depôt has been exceptionally fortunate, as the response to the first appeal was extraordinary, and since then a small steady stream of contributions has been maintained. The weekly contributions from workers produce a wonderful total, and some of the helpers have adopted original ways of adding to the funds. One lady makes and sells jig-saw puzzles, which produce a regular sum per week. Another sells cakes and recipes, while one makes artificial flowers for sale, and also takes photographs to be sold—the proceeds of which all go to our funds. One lady manages the tea-room, where, again, most of the articles are given by generous donors. A charge of 3d. per head is made for teas, and the lady in attendance does all the work in connection with that room, handing in her surplus money each week. One cannot conclude this article without paying a warm tribute to the splendid spirit of loyalty and goodwill which prevails throughout this depôt. Each individual worker seems imbued with just one idea, to do "as much as she can and as well as she can." It is, indeed, a fine record of women's work in Plymouth and the neighbouring country districts, and has been a useful example in organisation to many who have been anxious to do something for their country, and who needed some direction to fulfil that desire.

